# Necropsy Validation of a Novel Method for Left Ventricular Mass Quantification in Porcine Transthoracic and Transdiaphragmal Echocardiography

**DOI:** 10.3389/fcvm.2022.868603

**Published:** 2022-05-03

**Authors:** Charlotte Burup Kristensen, Stefan Michael Sattler, Anniek Frederike Lubberding, Jacob Tfelt-Hansen, Thomas Jespersen, Christian Hassager, Rasmus Mogelvang

**Affiliations:** ^1^Department of Cardiology, The Heart Center Rigshospitalet, Copenhagen, Denmark; ^2^Department of Biomedical Sciences, Faculty of Health and Medical Sciences, University of Copenhagen, Copenhagen, Denmark; ^3^Department of Forensic Genetics, Faculty of Health and Medical Sciences, University of Copenhagen, Copenhagen, Denmark; ^4^Department of Clinical Medicine, Faculty of Health and Medical Sciences, University of Copenhagen, Copenhagen, Denmark; ^5^Cardiovascular Research Unit, University of Southern Denmark, Svendborg, Denmark

**Keywords:** left ventricular mass, echocardiography, left ventricular hypertrophy, necropsy, animal model

## Abstract

**Introduction:**

Increased left ventricular mass (LVM) is one of the most powerful predictors of adverse cardiovascular events. Clinical evaluation requires reliable, accurate and reproducible echocardiographic LVM-quantification to manage patients. For this purpose, we have developed a novel two-dimensional (2D) method based on adding the mean wall thickness to the left ventricular volume acquired by the biplane method of disks, which has recently been validated in humans using cardiac magnetic resonance as reference value. We assessed the hypothesis that the novel method has better accuracy than conventional one-dimensional (1D) methods, when compared to necropsy LVM in pigs.

**Materials and Methods:**

Echocardiography was performed during anesthesia in 34 Danish Landrace pigs, weight 47–59 kg. All pigs were euthanized, cardiac necropsy was performed and the left ventricle was trimmed and weighed for necropsy LVM. Trans-thoracic echocardiography was applied for parasternal images. Transdiaphragmal echocardiography was applied for the apical images, which are otherwise difficult to obtain in pigs. We compared the conventional 1D- and 2D-methods and the novel 2D-method to the LVM from cardiac necropsy.

**Results:**

Necropsy LVM was 132 ± 11 g (mean ± SD). The novel method had better accuracy than other methods (mean difference ± 95% limits of agreement; coefficients of variation; standard error of the estimate, Pearson's correlation). Novel (−1 ± 20 g; 8%; 11 g; *r* = 0.70), Devereux (+26 ± 37 g; 15%; 33 g; *r* = 0.52), Area-Length (+27 ± 34 g; 13 %; 33 g; *r* = 0.63), Truncated Ellipsoid (+10 ± 30 g; 12%; 19 g; *r* = 0.63), biplane endo-/epicardial tracing (−3 ± 2 g; 10%; 14 g; *r* = 0.57). No proportional bias in linear regression was detected for any method, when compared to necropsy LVM.

**Conclusion:**

We confirm high accuracy of the novel 2D-based method compared to conventional 1D/2D-methods.

## Introduction

Increased left ventricular mass (LVM) is a well-known strong and independent predictor of adverse cardiovascular events and sudden death (1–4). The natural adaptive mechanisms of almost every cardiac condition are reflected in the degree of left ventricular (LV) hypertrophy. Increased LVM is associated with LV fibrosis (5) and reduction in LVM by blood pressure management (6) or valvular surgery (7) is associated with better outcome (6). For this reason, LVM has the potential to be used as a prognostic marker to detect clinical deterioration and may facilitate decision making for clinicians. Unfortunately, recommended conventional one-dimensional (1D) linear echocardiographic methods are less accurate and not suited for individual usage (8). Consequently, LVM quantification is often not performed or ignored and not included in the individual clinical decision-making.

We have recently presented a novel two-dimensional (2D) method based on adding the mean wall thickness to the left ventricular volume acquired by the biplane method of disks (9). The method is validated in humans using cardiac magnetic resonance (CMR) as gold standard. Furthermore, it is simpler but still as accurate as three-dimensional (3D) echocardiography and compared to the other 1D/2D/3D-methods it performs better regardless of LV geometry. The novel 2D-based method also demonstrated better reproducibility compared to the other methods, which is necessary for detection of small differences that may indicate early signs of deterioration.

Our aim was to assess whether the novel method has better accuracy than conventional 1D methods, when compared to necropsy LVM in pigs.

## Materials and Methods

### Study Population

We included thirty-four female Danish Landrace pigs, weight range 47–59 kg. All pigs were part of a project investigating arrhythmias during myocardial infarction and echocardiography was performed as part of the protocol (10, 11). The experiments were performed under the animal license number (2015-15-0201-00613) authorized by the Danish Animal Inspectorate in accordance with EU legislations for animal protection and care.

### Procedure

The pigs were premedicated, intubated and ventilated and anesthesia was maintained with continuous propofol infusion of 12.5 mg/h/kg (Propolipid 10 mg/ml, Fresenius Kabi AB, Uppsala, Sweden) and fentanyl 5 μg/h/kg (Fentanyl-Hameln 50 μg/ml, Hameln, Germany). Echocardiography was performed at baseline after placing a pulmonary artery catheter but before any other intervention. As part of the initial protocols, myocardial infarction was induced by balloon occlusion of the left anterior descending (LAD) artery just after the take-off of the first diagonal (D1) branch for 60–120 min and electrophysiological outcomes were studied. Pigs were euthanized at the end of the procedure by inducing ventricular fibrillation (VF) *via* burst pacing (50 Hz, 3 s, 7 V output) and cardiac necropsy was performed minutes after.

### Echocardiographic Acquisition and Analysis

**Figure 1:**
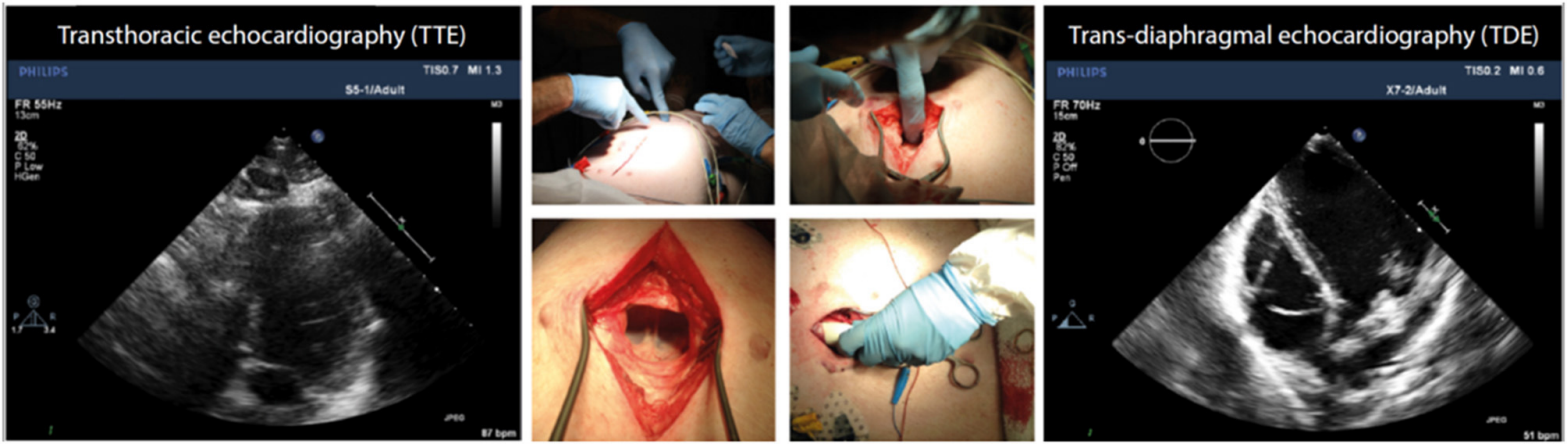
Trans-thoracic and trans-diaphragmal echocardiographic approach (12).

**Figure 2:**
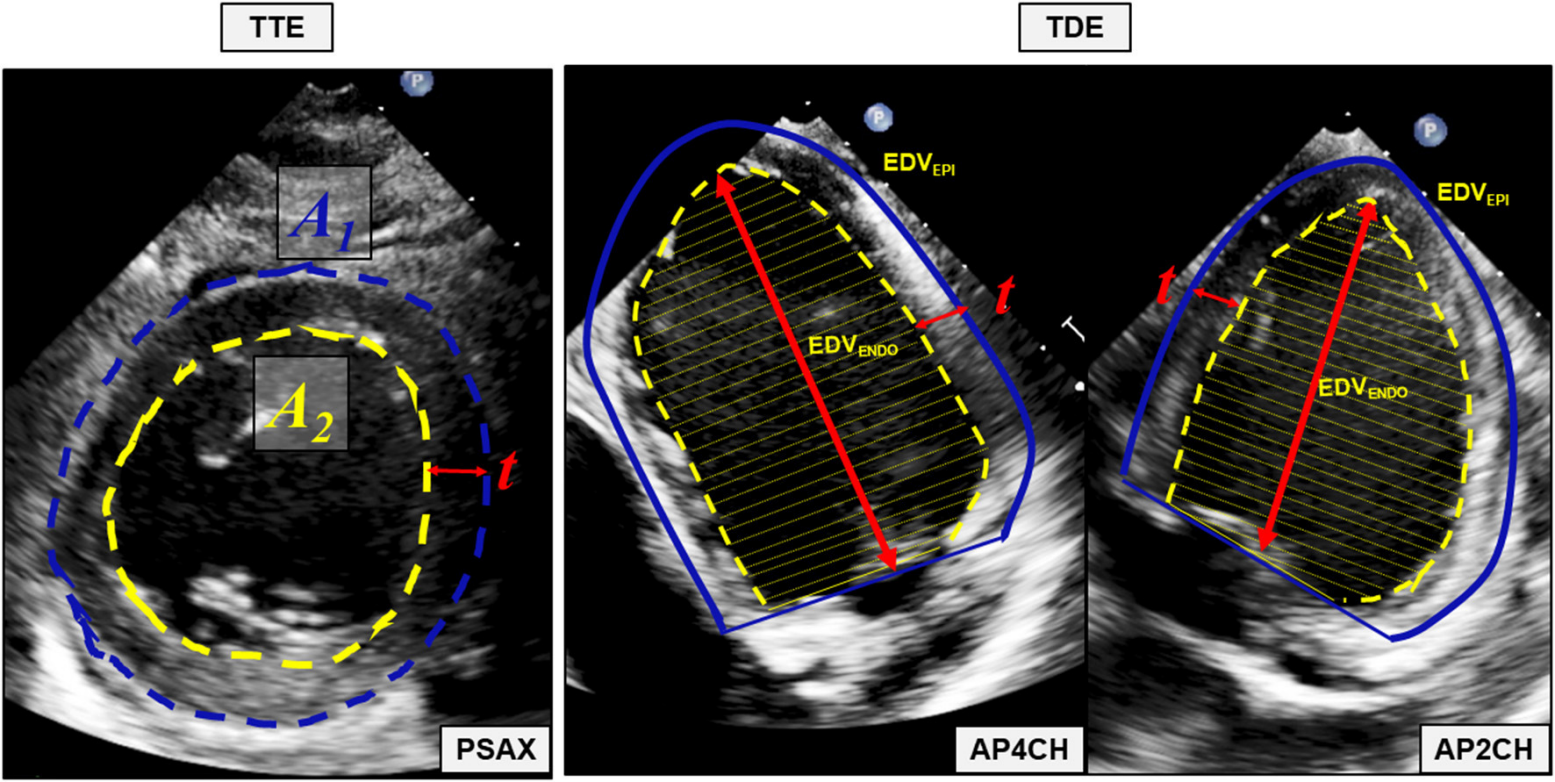
The novel 2D-based echocardiographic method for quantification of left ventricular mass. Mean wall thickness (*t*) is calculated by tracing of the endocardium (A_2_) and the epicardium (A_1_) in the parasternal short axis (PSAX) view. The left ventricular volume defined by the endocardium (EDV_ENDO_) is acquired using the biplane methods of disks with tracings of the endocardium in the apical four chamber (AP4CH) and apical two-chamber (AP2CH) view. Mean wall thickness (*t*) is added to each unique disk and a new volume, the left ventricular volume defined by the defined by the epicardium (EDV_EPI_) is quantified. Myocardial volume is calculated by subtracting EDV_ENDO_ from EDV_EPI_ and left ventricular mass is quantified by multiplying the myocardial volume with the myocardial density/gravity of 1.05 g/ml. **TTE** transthoracic echocardiography, **TDE**, transdiaphragmatic echocardiography; **PSAX**, parasternal short axis view; **A**_**1**_, Area defined by the epicardium; **A**_**2**_, Area defined by the endocardium; **t**, mean wall thickness; **AP4CH**, apical four chamber view; **EDV**_**ENDO**_, the left ventricular volume defined by the endocardium; **EDV**_**EPI**_, the left ventricular volume defined by the epicardium; **AP2CH**, apical two-chamber view.

Echocardiographic examinations were performed using an iE33 Echocardiography System scanner (Philips Medical Systems Nederlands B.V., Best, The Netherlands) with the pigs in supine position. The protocol for echocardiographic assessment included four views; the parasternal long-axis view (PLAX), the parasternal short-axis view (PSAX), the apical four-chamber view (A4CH) and the apical two-chamber view (A2CH). PLAX and PSAX were acquired by trans-thoracic echocardiography (TTE) using the S5-1-xMATRIX array transducer (1–5 MHz). A4CH and A2CH were acquired by trans-diaphragmal echocardiography (TDE) (Figure 1) through a small midline incision distal of the sternal xiphoid (12). The TDE approach was necessary to achieve appropriate images as the heart is aligned differently in the thorax of pigs compared to humans (13). For the apical views we used the Pediatric X7-2-xMATRIX array transducer (2–7 MHz) and because of limited space in the acoustic window we applied electronic rotation function *iRotate*, instead of physical rotation of the probe. All images were transferred as Digital Imaging and Communications in Medicine (DICOM) files to a local workstation and analyzed using the software EchoPAC Version: 203 Revision: 66.0 (GE Healthcare Vingmed Ultrasound, Horten, Norway). End-diastole was defined as the first frame with closure of the mitral valve. End-systole was defined as the frame with the smallest LV volume. End-diastolic volume (EDV), end-systolic volume (ESV) and left ventricular ejection fraction (LVEF) was evaluated by the 1D Teichholtz method (14) and by the 2D biplane methods of disks (8). We evaluated five different methods for LVM quantification, all presented in Table 1. All LVM-measurements were made at end-diastole. The electrocardiogram (ECG) was applied as reference in PLAX to find the corresponding frame in PSAX, where the mitral valve is not fully visible. Three of the methods; Devereux (15), Truncated Ellipsoid (TE) (16) and Area-Length (A-L) (17) are well-recognized and described in the current echocardiographic guidelines (8). For the biplane tracing of the endo- and epicardium we traced both the endo- and the epicardium, subtracted the inner volume (tracing of the endocardium) with the outer volume (tracing of the epicardium) to achieve the myocardial volume. The novel method (Figure 2) recently described (9) is validated in humans using CMR. The method adds the LV wall thickness from PSAX to EDV acquired by tracing of the endocardium in the biplane model, i.e., no tracing of the epicardium is necessary. The myocardial density/gravity of 1.05 g/ml was applied to convert myocardial volume to LVM. All measurements were performed by one reader (CK). Intra-reader analysis was performed by the same reader (CK) on same recordings and compared to the baseline measurements. Inter-reader analysis was performed by another reader (SS) on the same recordings and compared to the baseline measurements. For intra/inter-reader variability we only included pigs who had feasible measurements for all methods.

**Table 1:** Methods for left ventricular mass quantification.

**Method type**	**Method name**	**TTE**	**TDE**
1D	Devereux	PLAX	–
2D	Truncated Ellipsoid	PSAX	AP4CH
2D	Area-Length	PSAX	AP4CH
2D	Biplane tracing of the endo- and epicardium	–	AP4CH + AP2CH
2D	Novel	PSAX	AP4CH + AP2CH

*TTE, trans-thoracic echocardiography; TDE, trans-diaphragmal echocardiography; 1D, one-dimensional; 2D, two-dimensional; PLAX, parasternal long-axis; PSAX, parasternal short-axis; AP4CH, apical four-chamber; AP2CH apical two-chamber*.

### Necropsy

At the end of the experiment, ~10 min after VF was induced, a midline sternotomy was performed, the pericardium was removed and the heart including both ventricles and atria was explanted. If present, epicardial fat (which in general is scarce in young, lean pigs) and soft tissue was removed, and total heart weight measured. For measuring LVM the free wall of the right ventricle as well as valves, atria, papillary muscles and blood clots were removed. We used a commonly available digital scale (Wedo Electronic Precision Scale Optimo 1000, Werner Dorsch GmbH, Dieburg, Germany).

### Statistics

Statistical data analysis was performed in SPSS v25.0 (IBM Corp. IBM SPSS Statistics for Windows, Version 25.0. Armonk, NY). LVM-quantifications were performed in Windows Excel 2010 (Microsoft Office Professional Plus). Continuous variables expressed as mean and standard deviation (SD) and categorical values expressed as frequencies and percentage. Correlation was evaluated using Pearson's r. The accuracy was evaluated by paired *t*-test and presented as mean difference (bias) and 95% limits of agreement (LOA). The variations were expressed as the standard error of the estimate (SEE) and as coefficients of variation (CV) in percent and we adjusted the CV according to the anatomical LVM from necropsy. In the same manner we plotted the differences according to necropsy LVM. We decided to use this approach because we considered the necropsy LVM as the true value and not a method for comparison. Proportional bias was evaluated by linear regression with the necropsy LVM as independent variable and mean difference as dependent variable. Intra- and inter-reader variability was expressed as bias, LOA, SEE and CV compared to the baseline measurements. *P*-values <0.05 were considered statistically significant.

## Results

The baseline characteristics for the pigs are presented in Table 2. The total mass of the hearts by necropsy ranged from 200 to 295 g and the necropsy LVM ranged from 110 to 155 g. The hearts were relatively uniform in geometry and no pig had significant valve disease. Induced infarcts affected 15–20% of LV by visual inspection. LVEF by the biplane model of disks was 62 ± 6% (mean ± SD) and ranged from 47 to 74%.

**Table 2:** Baseline characteristics.

	**Mean ±SD**	**Range**
Pig weight (kg)	52 ± 2	47–59
Total heart weight by necropsy (g)	241 ± 22	200–295
Necropsy LVM (g)	132 ± 11	110–155
SBP (mmHg)	131 ± 16	88–169
DBP (mmHg)	82 ± 15	41–113
Heart rate (bmp)	81 ± 15	50–113
MWTd PLAX (cm)	0.93 ± 0.08	0.8–1.2
MWTd PSAX (cm)	0.97 ± 0.08	0.8–1.2
LVIDd (cm)	4.8 ± 0.3	4.4–5.4
LVIDs (cm)	3.2 ± 0.4	2.7–4.0
EDV Teichholtz (ml)	110 ± 14	89–141
EDV Biplane (ml)	97 ± 18	71–130
ESV Teichholtz (ml)	42 ± 12	27–71
ESV Biplane (ml)	37 ± 9	22–56
LVEF Teichholtz (ml)	61 ± 10	38–77
LVEF Biplane (ml)	62 ± 6	47–74

*SD, standard deviation; LVM, left ventricular mass; SBP, systolic blood pressure; DBP, diastolic blood pressure; MWTd, mean wall thickness diastole; PLAX, parasternal long-axis; PSAX, parasternal short-axis; LVIDd, left ventricular internal diameter diastole; LVIDs, left ventricular internal diameter systole; EDV, end-diastolic volume; ESV, end-systolic volume; LVEF, left ventricular ejection fraction*.

**Figure 3:**
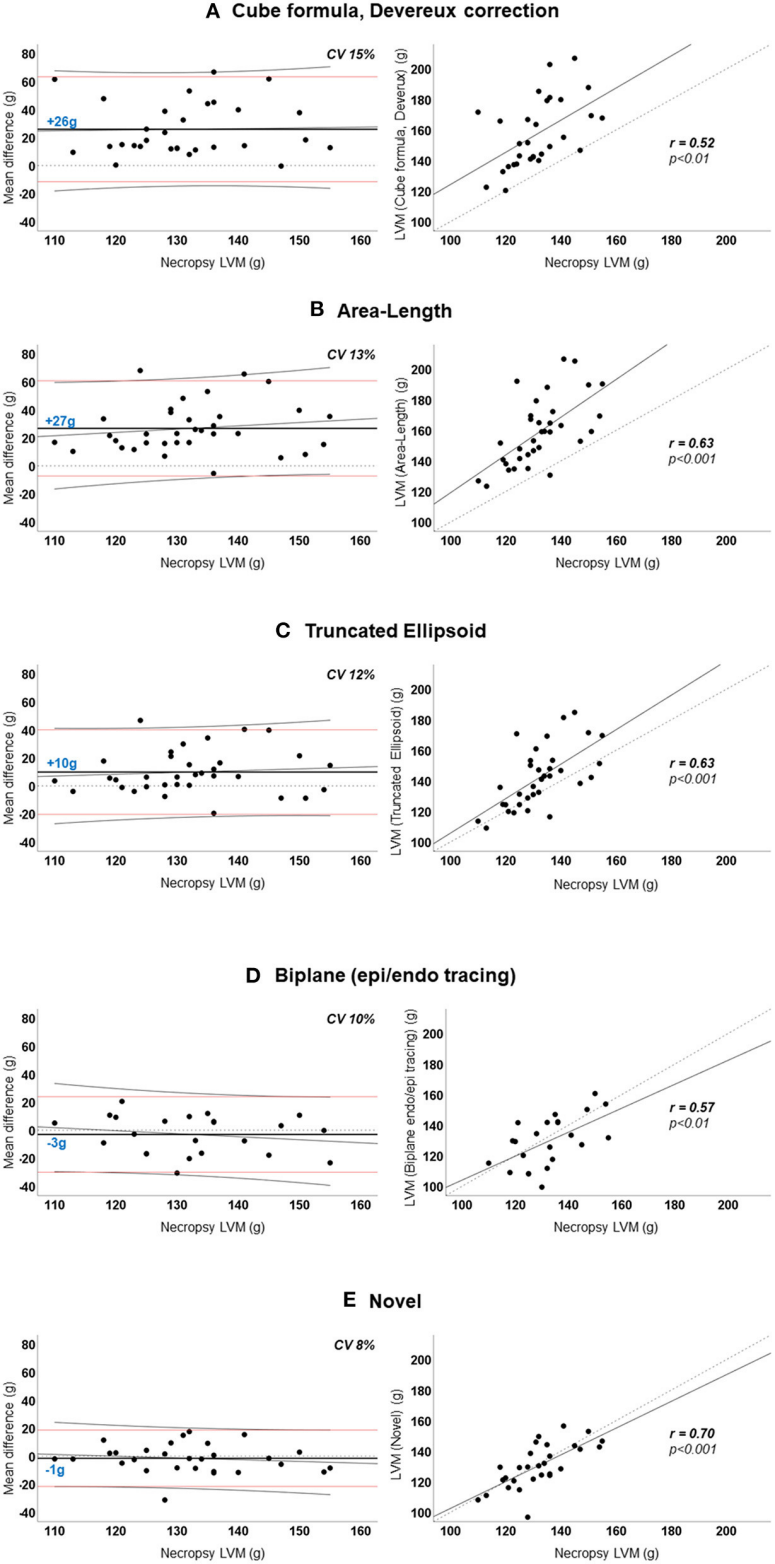
**(A–E)** Agreement between echocardiographic left ventricular mass and necropsy left ventricular mass. *Left panel:* Agreement between mean difference (echocardiographic-LVM – necropsy-LVM) and necropsy-LVM. Horizontal dotted black line indicates 0 (no difference). Horizontal solid black line and blue number is the mean difference; positive value indicates overestimation by echocardiography. Horizontal red lines are the 95% limits of agreement. Diagonal thin black line is the regression line with 95% confidence interval visualizing the proportional bias. *Right panel:* Linear regression curves of echocardiographic-LVM and necropsy-LVM with Pearson's correlation coefficient (r). Diagonal dotted line is the reference line. **LVM**, left ventricular mass; **CV**, coefficient of variation.

Figure 3 demonstrated the mean difference between the quantified LVM and the necropsy LVM (y-axis) according to the necropsy LVM (x-axis) (left panel) and correlation for LVM quantified by echocardiography (y-axis) according to necropsy LVM (x-axis) (right panel) for the various methods for the whole population. The novel model presented the lowest mean difference and the smallest variation than any of the other methods; CV was 8% compared to 10–13% for the other 2D-methods and 15% for the cube formula by Devereux. No method presented significant proportional bias in linear regression. The novel method had the best correlation to necropsy LVM; Pearson's *r* = 0.70, *p* < 0.001 followed by A-L and TE, both *r* = 0.63, *p* < 0.001. The results for all pigs (*n* = 34) are also presented in Table 3A.

**Table 3A:** Accuracy of various methods for left ventricular mass quantification among all pigs (*n* = 34).

					**Pearson's**	
	**Mean ±SD (g)**	Bias^*^±95%LOA (g)	**CV (%)**	**SEE (g)**	**r**	***p***
Necropsy left ventricular mass	132 ± 11					
Devereux	157 ± 22	+26 ± 37	15	33	0.52	*<0.01*
Area-Length	159 ± 22	+27 ± 34	13	33	0.63	*<0.001*
Truncated Ellipsoid	142 ± 20	+10 ± 30	12	19	0.63	*<0.001*
Biplane tracing of the endo-/epicardium	130 ± 16	−3 ± 27	10	14	0.57	*<0.01*
Novel	131 ± 14	−1 ± 20	8	11	0.70	*<0.001*

*Accuracy evaluated with necropsy left ventricular mass as reference value. SD, standard deviation, bias mean difference; LOA, limits of agreement; CV, coefficients of variation; SEE, standard error of the estimate; r, Pearson's correlation coefficient*.

* *Positive value indicates overestimation of left ventricular mass by echocardiography compared to left ventricular mass by necropsy*.

**Figure 4:**
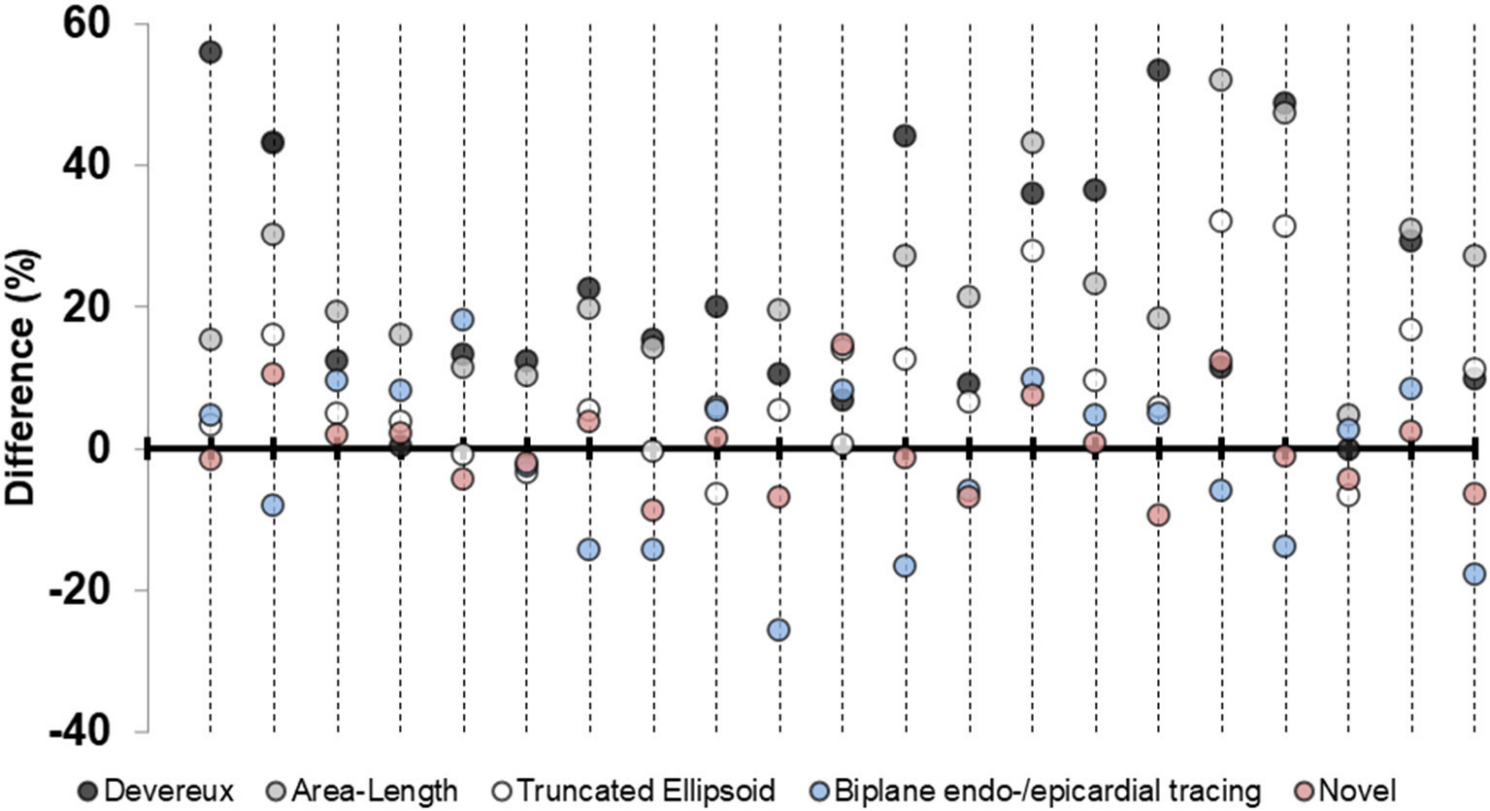
Percentage differences in left ventricular mass plotted for each pig. Differences in percent for the five echocardiographic methods for left ventricular mass-quantification for the subgroup of pigs (*n* = 21) where all measures were available. Positive value indicates overestimation by echocardiography compared to necropsy left ventricular mass. Each longitudinal line represents one pig.

We performed a subgroup analysis of the pigs where it was possible to quantify LVM by all echocardiographic methods (*n* = 21). The results for this subgroup are presented in Table 3B. The percentual difference between echocardiographic LVM and necropsy LVM was plotted in the y-axis for each pig (Figure 4). The novel method was the most accurate method for 11 (52%) of the pigs, followed by TE and biplane tracing of the endo-/epicardium, both 4 (19%) of the pigs, respectively. Devereux was the least accurate method for 10 (48%) pigs followed by A-L for 7 (33%) of the pigs.

**Table 3B:** Accuracy of various methods for left ventricular mass quantification among the pigs with 100% feasible measurements (*n* = 21).

					**Pearson's**	
	**Mean ±SD (g)**	Bias^*^±95%LOA (g)	**CV (%)**	**SEE (g)**	**r**	***p***
Necropsy left ventricular mass	131 ± 12					
Devereux	159 ± 24	+28 ± 42	16	37	0.46	*<0.05*
Area-Length	159 ± 24	+27 ± 32	12	33	0.80	*<0.001*
Truncated Ellipsoid	142 ± 22	+10 ± 28	11	18	0.81	*<0.001*
Biplane tracing of the endo-/epicardium	129 ± 16	−3 ± 28	11	15	0.53	*<0.05*
Novel	132 ± 14	0 ± 16	6	8	0.80	*<0.001*

*Accuracy evaluated with necropsy left ventricular mass as reference value. SD, standard deviation, bias mean difference; LOA, limits of agreement; CV, coefficients of variation; SEE, standard error of the estimate; r, Pearson's correlation coefficient*.

* *Positive value indicates overestimation of left ventricular mass by echocardiography compared to left ventricular mass by necropsy*.

Intra- and inter-reader variability is presented in Table 4 and we observe similar reproducibility as compared to the other 1D- and 2D-methods.

**Table 4:** Precision (reproducibility) of the various methods for left ventricular mass quantification among the pigs where all measures were available (*n* = 21).

	**Intra-reader variation**			**Inter-reader variation**		
	Bias^*^±95%LOA (g)	**CV (%)**	**SEE (g)**	Bias^*^±95%LOA (g)	**CV (%)**	**SEE (g)**
Devereux	9 ± 37	11.4	21.2	5 ± 32	10.0	17.4
Area-Length	0 ± 28	9.1	14.9	−10 ± 30	9.9	18.9
Truncated Ellipsoid	0 ± 25	8.9	13.0	−8 ± 26	9.8	16.3
Biplane tracing of the endo-/epicardium	−5 ± 23	9.2	13.0	−8 ± 21	8.8	13.9
Novel	3 ± 23	8.7	12.2	−9 ± 22	8.7	14.5

*SD, standard deviation, bias mean difference; LOA, limits of agreement; CV, coefficients of variation; SEE, standard error of the estimate; r, Pearson's correlation coefficient*.

* *Positive value indicates overestimation by intra- or inter-reader measurements*.

## Discussion

We demonstrate the accuracy of various echocardiographic methods for LVM-quantification in a porcine model with necropsy LVM as reference for LVM. The novelty of our approach is the study design combining necropsy validation with a novel echocardiographic method and improved imaging tools. The most important findings are the following:

1) The novel method for LVM-quantification is characterized by higher accuracy to LVM by necropsy and at least as good reproducibility as for the other 1D- and 2D-methods.
2) The conventional linear method by Devereux, which is recommended by current guidelines (8), overestimates LVM and demonstrates lower accuracy compared to the other methods.
3) We highlight an alternative way to increase echocardiographic apical image quality in animal models, by applying a trans-diaphragmatic approach and by applying the function *iRotate* since correct physical rotation of the probe may be difficult.

### Comparison to Previous Necropsy Validation Studies

Most studies with necropsy comparisons are published several decades ago and are naturally based on M-mode without 2D-guiding, or by less refined 2D echocardiographic technology. In 1979 Wyatt et al. (17) demonstrated better correlation and lower mean errors deploying 2D methods compared to linear methods and Salcedo et al. (18) demonstrated improved accuracy using 2D-methods. In 1986 Schiller et al. (19) confirmed high correlation and low SEE using 2D-methods. Two other studies by Woythaler et al. (20) in 1983 and Park et al. (21) in 1996 could not illustrate any improvement in 2D-methods compared to linear methods. On the contrary, some studies demonstrate excellent accuracy of linear methods among subjects with normal LV geometry (22, 23) and some studies unsatisfactory accuracy despite normal LV geometry (24). A direct comparison to these studies may be misleading compared to current imaging technology and improved post-processing software. A recent study by Miyashita et al. (25) validating LVM to necropsy values in pigs, demonstrates overestimation of LVM using linear methods as 2D-guided M-mode, in particular among the pigs with ischemic heart disease and LV dilatation. The same study also demonstrates a much narrower LOA using 2D-methods, which is in line with our results. The strength of our study compared to previous necropsy validation studies is that we have examined a variety of different methods on the same population, which makes it easier to compare the methods with each other instead of one-by-one in different populations.

### Aspects of Necropsy Validation as Reference for Left Ventricular Mass

What is the true value of LVM and how can it be measured? Previous studies have applied angiography, direct linear measures or echocardiography to validate LVM against cardiac autopsy in human models (15, 16, 20–24, 26–28) or cardiac necropsy in animal models (17–19, 29). Our validation methodology was to explant the heart of the pig immediately postmortem and to isolate the LV without papillary muscles and without any preservation. There are several aspects to consider when validating to the anatomical LVM by autopsy/necropsy. These are:

1) *The time between measurement and actual autopsy/necropsy*. Validation studies to human autopsy are usually limited by increased time duration between measurement and actual autopsy, whereas animal studies are usually validated at the same day, or at least preserved at the same day as the measurement by echocardiography was made. Human studies report durations of up to 454 days (28) between echocardiography and autopsy. As we performed necropsy immediately postmortem and at the same day as echocardiographic assessment, we were less limited by potential changes of the LV that may occur over time.
2) *The preservation methodology*. Formaldehyde or the aqueous solution of formaldehyde, also known as formalin, have often been applied as preservation methodology. This approach is reported to reduce LVM and volume without significant impact on myocardial density (30–32), whereas some studies report increased LVM after preservation (33). To overcome this potential limitation, several studies report adjustment of shrinkage by adding 5% to autopsy/necropsy LVM (23) or adjustment of increase by reducing autopsy/necropsy LVM by 2–3% (33). However, no adjustment at all (16, 24) is also reported. We did not use any preservation methodology and did not apply any adjustment to the weighed necropsy LVM.
3) *The autopsy/necropsy methodology*. Most studies report similar autopsy/necropsy methodology with removal of atria, right ventricle, epicardial fat and valves inspired by Geiser and Bove (16). In contrast to previous reports, we removed the papillary muscles from the myocardium. We claim that this approach is most truthful since the papillary muscles are excluded when LVM is measured by both echocardiography and CMR (9). We washed the hearts and removed visual blood clots and although we were very thorough, there may still be small clots left in the trabeculae or inside the vessels of the heart. The significance of this small amount of blood is probably negligible.
4) *Inhibition of the cardiac cycle in diastole or systole*. Cardiac arrest and eventually cardiac death was initiated by induced VF and consequently, most hearts were inhibited contracted, i.e., in systole. Inhibition in systole or diastole has impact on the volumes of the heart but should not affect the LVM significantly (16). This may even have minimized the effect of potential blood left in the vessels of the heart.
5) *Edema or fibrosis affecting the density/gravity of the LV*. Edema may be present in the ischemic region of the LV but also to some degree in adjacent and remote areas (34). During myocardial infarction, the density of the myocardium increases during initial edema and remains slightly higher after transition to fibrotic tissue compared to healthy myocardium (32). The pigs in our study were part of a protocol where myocardial infarction was induced by balloon occlusion of the LAD, which may have affected the density of the myocardium. Infarct sizes were determined to be within 15–20% of the LV myocardial mass.

### Necropsy Compared to Gold Standard Cardiac Magnetic Resonance

Necropsy validation has gradually been replaced by imaging modalities such as CMR, which is now considered the gold standard or reference method for left ventricular mass (35, 36). Excellent agreement between LVM by necropsy and by CMR-segmentation has been reported (37–40). Studies using CMR as reference method report overestimation of LVM by linear methods (41, 42) and better agreement for the 2D-methods (43). We Kristensen et al. (9) have demonstrated similar overestimation for the 1D-methods and better agreement for 2D-methods, especially the novel method. Also, reproducibility from the whole cohort and accuracy of the patients with normal geometry were very similar to the accuracy for the pigs in this cohort, which all had normal geometry as well.

### The Porcine and the Human Heart

The porcine heart is aligned differently in the thoracic cage compared to the human heart (13). In humans, the upright orientation of the body and the location of the heart in the thoracic cage, gives the heart a “trapezoidal shape” with its apex pointing downward to the left with an oblique angle to the long axis of the body. Since pigs walk on four legs and have differences in the shape of the thoracic cage, the heart is “Valentines heart” shaped with its apex pointing forward and perpendicular to the long axis of the body. In supine position, the heart will orientate with its axis pointing more downwards and toward the diaphragm. This facilitates parasternal images acquisition through transthoracic acquisition in pigs. However, correctly aligned apical views become more difficult to acquire. To be able to place the transducer as close to the apex of the heart as possible, we made a small incision below the sternum and placed the probe under the diaphragm for transdiaphragmatic acquisition, which improved the image quality significantly (12). Because of limited space within the incision and consequently difficulties in performing physical rotation of the probe, we also applied the *iRotate* application for optimal 4CH- and 2CH-view. We recommend this echocardiographic approach among research animals who are euthanized at the study day. Other potentially differences that have been described between the human and porcine hearts are a more conical morphology of the LV and the heart, coarser papillary muscles and thicker LV wall compared to humans (13). The wall thickness range in our cohort was 0.8–1.2 cm, which would be mildly abnormal if translated to human references (8).

### Limitations

The pigs in this cohort did not have any structural or congenital heart disease and the geometrical pattern of the LV was considered normal and uniform for all pigs. The results from this study may not be applicable to deviating LV geometries. All pigs were subjected to acute myocardial infarction affecting 15–20% of the LV. Affected myocardium might have had higher weight due to edema. As echocardiography was performed before myocardial infarction, this could have resulted in a systematic error. We were not able to perform 3D echocardiography due to technical limitations such as, difficulties in pausing the respirator, reverberations and artifacts disturbing the images. As the purpose of this study was to validate echocardiographic method on pigs with the intention of usage on humans, geometrical differences between porcine and human hearts must be kept in mind.

## Conclusions

We demonstrate necropsy validation in a porcine model of a recently presented 2D-based echocardiographic method for LVM-quantification. We confirm high accuracy of the novel 2D-based echocardiographic method compared to the other conventional 1D/2D echocardiographic methods.

## Data Availability Statement

The raw data supporting the conclusions of this article will be made available by the authors, without undue reservation.

## Ethics Statement

The animal study was reviewed and approved by the Danish Animal Inspectorate in accordance with EU legalisations for animal protection and care (animal license number 2015-15-0201-00613).

## Author Contributions

CK, SS, JT-H, and RM planned the study. CK, SS, and AL conceived the study. CK performed the echocardiographic examinations, the data analysis, and wrote the manuscript. SS contributed to the inter-observer analysis. TJ and CH provided critical feedback. All authors discussed the results and contributed to the manuscript.

## Funding

This work was funded by the Novo Nordisk Foundation Tandem Programme (TJ and JT-H), registered under Grant No. 31364.

## Conflict of Interest

The authors declare that the research was conducted in the absence of any commercial or financial relationships that could be construed as a potential conflict of interest.

## Publisher's Note

All claims expressed in this article are solely those of the authors and do not necessarily represent those of their affiliated organizations, or those of the publisher, the editors and the reviewers. Any product that may be evaluated in this article, or claim that may be made by its manufacturer, is not guaranteed or endorsed by the publisher.
